# Identification and Biopsy of Sentinel Lymph Node in Early-Stage Cervical Carcinoma: Diagnostic Accuracy and Clinical Utility

**DOI:** 10.7759/cureus.23838

**Published:** 2022-04-05

**Authors:** Ioanna Koutroumpa, Michail Diakosavvas, Maria Sotiropoulou, Vasilios Pergialiotis, Kyveli Angelou, Michalis Liontos, Dimitrios Haidopoulos, Aristotelis Bamias, Alexandros Rodolakis, Nikolaos Thomakos

**Affiliations:** 1 Division of Gynecologic Oncology, 1st Department of Obstetrics and Gynecology, Alexandra Hospital, National and Kapodistrian University of Athens, Athens, GRC; 2 Department of Pathology, Alexandra Hospital, National and Kapodistrian University of Athens, Athens, GRC; 3 Oncology Unit, Department of Clinical Therapeutics, Alexandra Hospital, National and Kapodistrian University of Athens, Athens, GRC; 4 2nd Propaedeutic Department of Internal Medicine, Attikon Hospital, National and Kapodistrian University of Athens, Athens, GRC

**Keywords:** sentinel, methylene blue, lymph node biopsy, lymphatic mapping, uterine cervical cancer

## Abstract

Background

Due to the subsequent complications of pelvic lymphadenectomy in patients with early-stage cervical cancer, the sentinel lymph node (SLN) technique has been increasingly employed. This study aimed to investigate the detectability of SLN using methylene blue and explore the diagnostic accuracy of SLN biopsy.

Methodology

A study was conducted from September 2015 to August 2018 and included 90 women with cervical cancer, FIGO (International Federation of Gynecology and Obstetrics-2009) stage IA1-IIA1. Methylene blue was injected intracervically. Any detected dyed nodes were sent for frozen section biopsy, followed by bilateral pelvic lymphadenectomy. The predictive ability of SLN was evaluated in statistical terms after comparison of intraoperative biopsy and final histopathology.

Results

The sensitivity, specificity, false-negative rate, positive predictive value, and negative predictive value (NPV) were 55.6%, 95.1%, 4.9%, 55.6%, and 95.1%, respectively. The SLN performance in patients with tumor size ≤2.2 cm, negative lymphovascular space involvement, and depth of stromal invasion ≤5 mm was superior (sensitivity 100%, specificity 93.5%, NPV 100%).

Conclusions

The SLN technique with blue dye alone is a feasible and adequate alternative to systematic lymphadenectomy in early-stage cervical cancer in selected patients, given that a strict algorithm is applied.

## Introduction

Traditionally, the standard of care regarding the surgical treatment for women with early-stage cervical cancer included radical hysterectomy (or radical trachelectomy in case of fertility-sparing surgery) and assessment of the status of pelvic lymph nodes by complete pelvic bilateral lymphadenectomy [1,2]. Currently, imaging techniques such as magnetic resonance imaging (MRI) and positron emission tomography have been integrated into the preoperative evaluation of pelvic and para-aortic nodal involvement. Nonetheless, in many cases, these examinations are not accurate enough, with sensitivity varying from 56% to 75%; thus, to date, the surgical approach remains the gold standard for nodal assessment [2-6].

However, lymph node dissection is associated with numerous complications such as extensive bleeding, formation of lymphocysts, lymphedema, nerve and ureteral injuries, infections, and thromboembolism [1,3,7-10]. Considering that nodal involvement is present only in 15-20% of early-stage cervical cancer cases, it has been estimated that more than 80% of these patients undergo an unnecessary and possibly risky surgical procedure such as pelvic lymphadenectomy [1,3,8,9]. In an effort to overcome the above-mentioned complications, the sentinel lymph node (SLN) technique has emerged as a promising alternative method to lymphadenectomy for patients with uterine and cervical carcinoma [8,11]. During the past few years, many types of dyes and tracers have been investigated as possible markers of SLNs and various techniques have been employed with different diagnostic accuracy and effectiveness. However, many of these new technologies despite being very effective are costly and require special equipment, and hence are not widely available [11,12]. In this study, we aimed to investigate the detectability of SLN using methylene blue in patients with early-stage cervical carcinoma and explore the diagnostic accuracy of SLN biopsy.

## Materials and methods

A prospective study including women with cervical cancer, FIGO (International Federation of Gynecology and Obstetrics) stage IA1-IIA1 (Clinical Stages 2009), was conducted from September 2015 to August 2018. During this period, consecutive patients with histologic confirmation of cervical cancer who were admitted to the gynae/oncology department of our hospital and met the inclusion criteria were included in the study. To allow for homogeneity and establish the learning curve of the process faster, every procedure included in the study was performed by the members of the same surgical team, which consisted of four gynae/oncology surgeons. The study was designed considering the Declaration of Helsinki concerning human rights. All patients signed informed consent, and the study was registered and approved by the scientific board and ethical committee of our institution (IRB number: 86/12.2.2015).

Inclusion criteria were (a) primary early-stage cervical cancer FIGO 2009 stage IA1-IIA1 verified by histopathology, (b) tumor size ≤4 cm (diagnosed preoperatively), and (c) performance status adequate for extensive surgical procedures. Preoperatively, all patients underwent MRI examination. Patients with advanced disease with parametrial invasion, distant metastasis, hydronephrosis, or suspicious enlarged pelvic or para-aortic nodes on the preoperative clinical or imaging evaluation were excluded. Moreover, patients allergic to blue dye, those with previous major operations in the pelvis, recurrent cervical cancer, and a history of pelvic radiotherapy were also excluded. In case of not being able to identify any SLNs in a patient during the surgical operation, either blue-dyed or enlarged palpable nodes, the procedure was characterized as a failed lymph node mapping, and the patients were excluded from the study and treated according to the institution’s protocols.

On the day of the surgery, after the induction of anesthesia, intracervical injection of methylene blue dye was performed under direct visualization at the three and nine o’clock sites of the cervix using a 22-gauge spinal needle. A total of 4 mL of dye was used, of which 1 mL was injected superficially and 1 mL deep. The surgical procedure started 10 minutes after the completion of the injection to allow for the tracer to flow toward the lymphatic drainage of the cervix and dye the pelvic nodes.

The retroperitoneum was opened on both with monopolar diathermy to avoid any bleeding that would compromise the identification of blue lymphatic channels and nodes. If a blue lymphatic channel was visualized, it was followed until a blue node was found. Any detected blue nodes as well as any palpably enlarged nodes after the exploration of the retroperitoneum were considered SLNs and were extracted and sent for intraoperative frozen section biopsy. Afterward, a complete bilateral pelvic lymphadenectomy was performed, followed by the completion of the operation according to the institution’s protocols (simple extrafascial or radical hysterectomy), and the specimens were sent for pathologic examination. In the case of a positive intraoperative biopsy on SLN, the surgeon had to decide whether to continue and complete or abandon the surgical procedure. SLN mapping was considered successful if at least one SLN was identified. When the frozen section biopsy of the SLN was negative but the final histopathology reported a metastatic non-SLN, the SLN procedure was defined as a false negative.

All SLNs that were sent for intraoperative biopsy were examined by hematoxylin and eosin staining. SLNs were examined again in the final pathologic evaluation of the specimens. Ultrastaging of SLNs was not performed. In the final histopathologic assessment, all nodes including the SLNs were examined separately and thoroughly by two experienced pathologists in gynecological cancer. The results of the intraoperative biopsy were compared to the final histopathology. The rest of the specimens, such as the uterus, that were sent for pathologic evaluation were examined according to our institution’s protocols.

**Figure 1:**
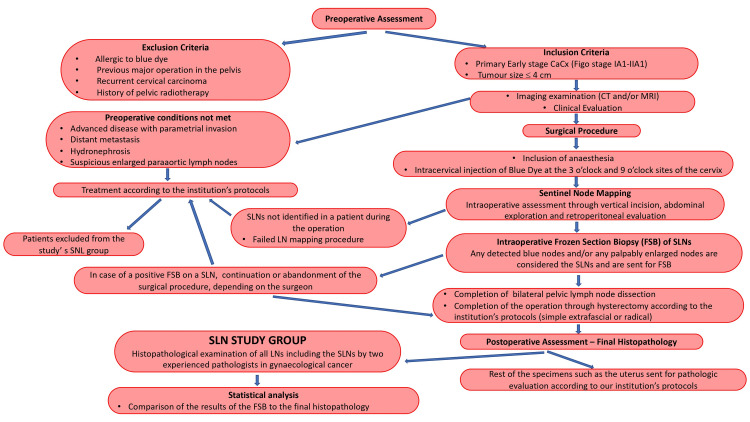
Study algorithm. CaCx: cervical cancer; FIGO: International Federation of Gynecology and Obstetrics; CT: computerized tomography; MRI: magnetic resonance imaging; SLN: sentinel lymph node; LN: lymph node; FSB: frozen section biopsy

Quantitative variables are expressed as mean and standard deviation (SD) and/or as median and interquartile range (IQR). Qualitative variables are expressed as absolute and relative frequencies. The predictive ability of SLN was evaluated by calculating sensitivity, specificity, and positive and negative predictive values. All p-values reported are two-tailed. Statistical significance was set at 0.05, and analyses were conducted using SPSS software version 23 (IBM Corp., Armonk, NY, USA). The analysis was performed on a per-patient basis and not per side of lymphadenectomy (Figure 1).

## Results

The study sample consisted of 90 women with a mean age of 49.0 years (SD = 9.4 years). The mean follow-up duration was 33 months (SD = 17.4 months), and the median follow-up duration was 35.5 months (IQR = 18-47 months). None of the patients died during follow-up. The depth of stromal invasion was more than 5 mm in 44 (48.9%) cases, and lymphovascular space invasion was positive in 35 (38.9%) cases. A history of cervical conization was present in 41.1% of the sample. The majority of the women (95.6%) had a radical hysterectomy (Type II and III, Piver and Rutledge Classification), while one patient underwent a simple extrafascial hysterectomy (Type I, Piver and Rutledge Classification). The histological type was mostly squamous carcinoma (65.6%), followed by adenocarcinomas (28.9%). Based on FIGO 2009 staging, 11.2% of the patients had stage IA, 84.4% of the women had stage IB1, and 4.4% had stage IB2-IIA1 (Table 1).

**Table 1:** Demographics and clinical characteristics. ^¶^Types of radical hysterectomy, Piver and Rutledge Classification. ^ⱡ^FIGO staging, Clinical Stages of Cervical Cancer (2009). SD: standard deviation; LVSI: lymphovascular space invasion

	N = 90 (%)
Age (years), mean (SD)	49.0 (9.4)
Maximum tumor size (cm), mean (SD)	2.3 (1.2)
Depth of stromal invasion	
≤5 mm	46 (51.1)
>5 mm	44 (48.9)
LVSI	
Negative	55 (61.1)
Positive	35 (38.9)
History of cervical conization	
No	53 (58.9)
Yes	37 (41.1)
Type of surgery	
Radical hysterectomy (Type II and III)^¶^	86 (95.6)
Simple extrafascial hysterectomy (Type I)^¶^	1 (1.1)
Other	3 (3.3)
Histological type	
Squamous	59 (65.6)
Adenocarcinomas	26 (28.9)
Adenosquamous	1 (1.1)
Other	4 (4.4)
Stage^ⱡ^	
IA1	5 (5.6)
IA2	5 (5.6)
IB1	76 (84.4)
IB2	3 (3.3)
IIA1	1 (1.1)

The mean number of SLN found was 2.5 (SD = 1.3) and ranged from one to eight, while the mean number of pelvic lymph nodes removed was 27.4 (SD=10.8) and ranged from 11 to 63. Metastatic disease to nodes was recorded in nine (10.0%) cases, and nine (10.0%) of the patients had a positive intraoperative biopsy for SLN. Regarding the results from SLN mapping, in 88.9% of the sample, SLN was found bilaterally, and in 11.1% of the sample, SLN was found unilaterally. The most common mapping position of SLNs were the external iliac lymph nodes (55.6%), followed by the internal iliac nodes (12.2%) and the interiliac nodes at the bifurcation of the iliac vessels (11.3%), whereas 6.7% were found in unexpected areas (parametrium). All cases with parametrial SLNs were detected in patients with tumor sizes of 2.2 cm or more, positive lymphovascular space invasion, and depth of stromal invasion of 5 cm or more. Parametrial involvement was not detected when SLNs were negative (Table 2).

**Table 2:** SLN mapping, FSB results, and final histology of LNs. SLN: sentinel lymph node; FSB: frozen section biopsy; LN: lymph node; SD: standard deviation

	N (%)
SLN detection	
Unilateral	10 (11.1)
Bilateral	80 (88.9)
SLN mapping position	
External iliac	108 (55.6)
Internal iliac	26 (12.2)
Iliac bifurcation - interiliac	24 (11.3)
Obturator fossa	22 (10.4)
Parametrial	14 (6.7)
Common Iliac	8 (3.8)
Number of SLN(s) found, mean (SD)	2.5 (1.3)
Number of total LN(s) removed, mean (SD)	27.4 (10.8)
Histology for LN	
Negative	81 (90.0)
Positive	9 (10.0)
Intraoperative biopsy for SLN	
Negative	81 (90.0)
Positive	9 (10.0)

SLN correctly predicted the presence or absence of positive lymph nodes in 82/90 cases, resulting in diagnostic accuracy of 91.1%. The sensitivity, specificity, false-positive rate (FPR), false-negative rate (FNR), positive predictive value (PPV), and negative predictive value (NPV) were 55.6%, 95.1%, 44.4%, 4.9%, 55.6%, and 95.1%, respectively (Table 3).

**Table 3:** Sentinel lymph node performance in all patients (N = 90). LN: lymph node; SLN: sentinel lymph node; PPV: positive predictive value; NPV: negative predictive value

Intraoperative biopsy for SLN	Histology for LNs		False-negative (%)	False-positive (%)	Sensitivity (%)	Specificity (%)	PPV (%)	NPV (%)
	Negative	Positive						
	N	N						
Negative	77	4	4.9	44.4	55.6	95.1	55.6	95.1
Positive	4	5						

The SLN performance in patients (N = 32) with tumor size ≤2.2 cm, negative lymphovascular space invasion, and depth of stromal invasion ≤5 mm was found to be superior in this group of women regarding sensitivity (100%), specificity (93.5%), NPV (100%), FNR (6.5%), and FPR (0%) (Table 4).

**Table 4:** Sentinel lymph node performance in patients with tumor size ≤2.2 cm, negative LVSI, and depth of stromal invasion ≤5 mm (N = 32). LN: lymph node; SLN: sentinel lymph node; PPV: positive predictive value; NPV: negative predictive value; LVSI: lymphovascular space invasion

Intraoperative biopsy for SLN	Histology for LN		False-negative (%)	False-positive (%)	Sensitivity (%)	Specificity (%)	PPV (%)	NPV (%)
	Negative	Positive						
	N	N						
Negative	29	0	6.5	0.0	100.0	93.5	33.3	100.0
Positive	2	1						

## Discussion

The findings of our study suggest that the diagnostic accuracy of SLN detection using blue dye in patients with small tumor size, absence of lymphovascular space invasion, and minimal depth of stromal invasion (≤5 mm) is excellent as the sensitivity and specificity of the method reached 100% and 93.5%, respectively. However, the results were not promising in our full cohort as the sensitivity of the method was 55.6%. The majority of SLNs were located along the iliac vessels, and just 6.7% of the vessels were found in the parametrium. Bilateral staining of SLNs was achieved in 89% of patients.

The detection rate and bilaterality of detection of SLNs that were observed in our whole cohort are comparable to that of previous studies published in the field of cervical cancer and depict the low sensitivity of the method, despite the fact that SLN harvesting (number of detected SLNs) is considered adequate [13,14]. The high diagnostic accuracy of methylene blue staining in patients with small lesions has not been reported previously in the international literature. However, a recent study conducted by Snyman et al. suggested that the presence of enlarged nodes (which were excised in our cohort as potentially malignant) was far more prevalent in patients with locally advanced-stage disease compared with early-stage disease (47.6% vs. 19.6%) [14]. Nodal metastasis was also more prevalent (47.6% vs. 15.7%). Interestingly, however, in this series, bilateral detection of SLNs was very low compared to ours (30.5%) and did not differ among the two aforementioned groups (28.6% vs. 31.4%; p = 0.816). In another study by Salvo et al., methylene blue alone performed worse in detecting bilateral lesions compared to our series (55% of cases) [1]. Stratification of cases per stage of the disease was comparable to ours, and, interestingly, the authors did not observe a significant effect of tumor size in the overall and bilateral detection rate of harvested nodes. However, the diagnostic accuracy of the method was not compared among patients with small and larger legions.

Blue dye injection for the detection of stained nodes with the naked eye (isosulfan, methylene, or patent blue) was the first described method in cervical cancer patients [15]. The advantages of blue dye are the low cost of the method because no additional equipment is required besides the dye [16]. Some drawbacks associated with the use of blue dye include the allergic reactions seen in less than 1% of the patients and the staining that it causes in the operative field, resulting in difficulties in SLN mapping [17]. Radioisotope technetium-99 (or 99mTc) has been described as a single agent or in comparison with methylene blue to increase the detection rate of the dye [18,19]. Comparing the use of both tracers, 99mTc and blue dye versus either one of them alone, most studies have found that the combined method of lymphatic mapping may result in an improvement in the detection rate of SLN(s) (99.1% vs. 93%; p = 0.009), while some others studies did not report the same conclusions [15,20]. In the last years, indocyanine green has been instituted in the field of SLN; however, its superiority compared to 99mTc and its combination with blue dye has been challenged in a previous meta-analysis [21]. Nonetheless, a significant increase in the overall and bilateral detection rate of SLN mapping has been demonstrated when indocyanine green is compared to blue dyes alone ﻿(odds ratio (OR) = 0.27; 95% confidence interval (CI) = 0.15-0.50; p < 0.0001) [21]. In the FILM trial that assessed the difference in the ability of SLN detection by different tracers, indocyanine green confirmed its superiority over ﻿the use of isosulfan blue dye solely. In a sample of 176 patients with 485 identified lymph nodes, 471 (97%) were detected with the use of the green dye, while only 226 (47%) nodes were found with the blue dye (difference = 50%, 95% CI = 39-62; p < 0.0001) [22]. A major factor that favors the use of indocyanine green is the ease of inspection of the parametrial and paracervical regions without impairing parametrial dissection [17]. Conversely, one of the main concerns regarding this method is the need for a dedicated optical filter and camera (﻿near-infrared fluorescence spectroscopy) because the staining is invisible to the human eye [16,23]. Thus, the cost of such equipment makes this technique significantly more expensive than the blue dye method.

Our study has certain advantages as well as limitations. The fact that all patients were managed by the same surgical team can be considered an advantage because the results extracted should illustrate more accurately the potential benefits of this procedure. A major limitation of our study is the fact that ultrastaging was not performed due to the inability of the histopathological department to support this demanding and time-consuming project because of limited human resources and heavy workload. Another limitation was the absence of 99m-Tc-nanocolloid use. The reason for this was the lack of the necessary equipment, as well as the higher cost of the procedure [16]. On the other hand, in our study, we describe for the first time in the international literature the high diagnostic accuracy of the method in patients with early-stage disease with an absence of lymphovascular space invasion and depth of invasion ≤5 mm.

This information should be further reviewed by future researchers as it may benefit the use of SLN in countries and institutions that lack the adequate financial background to support indocyanine green and/or 99mTc. Information concerning the depth of invasion retrieved by conization samples in patients with early-stage disease can be used in this direction. Ultrastaging techniques should be also instituted in these cases, and we believe that the cost-effectiveness of the method in selected patients may overcome the benefit of indocyanine green, although this field remains, to date, unexplored. Lastly, the performance of the technique in terms of postoperative complications and patient survival needs to be assessed in prospective randomized trials to assess the safety of the technique.

## Conclusions

Our series reinforces the findings of previous similar studies on the concept of SLN identification in the early stages of cervical cancer with the use of blue dye. Our results confirm the clinical importance of SLN analysis in minimizing systematic lymphadenectomy even with blue dye alone and support less radical surgical approach to the retroperitoneum with greater safety in patients with small tumors. Considering the use of blue dye alone as an SLN tracer, this study establishes our protocol as feasible, adequate, and relatively inexpensive in selected patients, especially in cases with minimal risk factors for nodal metastasis.
